# Barriers and Unequal Access to Timely Molecular Testing Results: Addressing the Inequities in Cancer Care Delays across Canada

**DOI:** 10.3390/curroncol31030103

**Published:** 2024-03-06

**Authors:** Stephanie Snow, Christine Brezden-Masley, Michael D. Carter, Neesha Dhani, Cassandra Macaulay, Ravi Ramjeesingh, Michael J. Raphael, Monika Slovinec D’Angelo, Filomena Servidio-Italiano

**Affiliations:** 1 Division of Medical Oncology, QEII Health Sciences Centre, Halifax, NS B3H 2Y9, Canada; 2 Division of Medical Oncology, Sinai Health, Toronto, ON M5G 1X5, Canada; 3 Division of Anatomical Pathology, QEII Health Sciences Centre, Halifax, NS B3H 2Y9, Canada; 4 Division of Medical Oncology & Hematology, Princess Margaret Cancer Centre, Toronto, ON M5G 2M9, Canada; 5 Colorectal Cancer Resource & Action Network (CCRAN), Toronto, ON M4W 3E2, Canada; 6 Odette Cancer Centre, Sunnybrook Health Sciences Centre, Toronto, ON M4N 3M5, Canada

**Keywords:** biomarker, biomarker testing, biomarker status, circulating tumor DNA (ctDNA), comprehensive genomic profiling, health systems, liquid biopsy, molecular testing, next generation sequencing, precision medicine

## Abstract

Genomic medicine is a powerful tool to improve diagnosis and outcomes for cancer patients by facilitating the delivery of the right drug at the right dose at the right time for the right patient. In 2023, a Canadian conference brought together leaders with expertise in different tumor types. The objective was to identify challenges and opportunities for change in terms of equitable and timely access to biomarker testing and reporting at the education, delivery, laboratory, patient, and health-system levels in Canada. Challenges identified included: limited patient and clinician awareness of genomic medicine options with need for formal education strategies; failure by clinicians to discuss genomic medicine with patients; delays in or no access to hereditary testing; lack of timely reporting of results; intra- and inter-provincial disparities in access; lack of funding for patients to access testing and for laboratories to provide testing; lack of standardized testing; and impact of social determinants of health. Canada must standardize its approach to biomarker testing across the country, with a view to addressing current inequities, and prioritize access to advanced molecular testing to ensure systems are in place to quickly bring innovation and evidence-based treatments to Canadian cancer patients, regardless of their place of residence or socioeconomic status.

## 1. Introduction

Cancer is the leading cause of death in Canada [1,2] and 45% of Canadians can expect to be diagnosed with cancer during their lifetime [3]. In 2021, cancer-related costs in Canada were estimated at USD 26.2 billion, 30% of which were borne by patients and their families [4].

According to the Pan-Cancer Analysis of Whole Genomes Consortium, up to 95% of tumors have identifiable mutations that are responsible for tumor proliferation (driver mutations). Some mutations occur before a tumor becomes invasive or metastasizes, with implications for early cancer detection and biomarker development [5]. Precision medicines are guided by a tumor’s biomarker status and represent a tremendous advancement in cancer treatment. However, systemic barriers to accessing timely biomarker testing, such as national funding for reimbursement, can limit effective precision cancer care. Cancer patient advocacy groups as well as healthcare professionals have identified challenges and opportunities for change at the education, delivery, laboratory, patient, and health-system levels, many of which are common across different tumor types. One such advocacy support network, the Colorectal Cancer Resource & Action Network (CCRAN) organized a biomarker conference to bring forward thought leaders across different tumor types to address these challenges and opportunities. Below are the highlights presented at the 2023 CCRAN Biomarker Conference.

## 2. Biomarker Conference

### 2.1. Impetus and Organization

The conference was organized under the auspices of CCRAN, a not-for-profit organization that, through its advocacy, education, and research initiatives, strives to address challenges that impede optimal delivery of cancer care and patient outcomes in Canada. Their work with patients and caregivers brought to light the incomplete and inconsistent knowledge patients have about their own access to biomarker testing, specifics of what has (or has not) been tested, and of their own results. Further, the national scope of CCRAN highlighted the variability in access to both biomarker testing and precision medicine, within and between health jurisdictions in Canada.

CCRAN reached out to other cancer patient advocacy groups and found that all groups identified similar barriers, challenges, and concerns with regards to biomarker testing. This finding was the impetus for this conference. Directions on the conference objectives, agenda, speakers, and participants were provided by the conference steering committee (see Acknowledgments). The conference was held virtually on 21 and 22 June 2023, with a total of 361 registrants. Audience members were able to interact with presenters using the chat feature for questions. The conference agenda and speakers are presented in Appendix A and session recordings are available at: https://videos.ccran.org/Biomarkers2023/ (accessed on 1 March 2024). A glossary of terms used across conference presentations is presented in Appendix B.

This report summarizes the highlights of the conference discussions and recommendations for action that were distilled by the authors based on a review of the content of expert presentations delivered during the conference and of the ensuing discussions held during each session. No processes such as a Delphi methodology were employed during the conference to formally derive consensus opinions.

### 2.2. Objectives

The objectives of the conference were to highlight: (1) cancer patient experiences related to accessing molecular testing and precision medicines in Canada; (2) the role of timely biomarker testing and reporting in medical-treatment decision making; (3) health-system preparedness for equitable access to advanced molecular testing; and (4) the value of timely and personalized diagnostic approaches in optimizing cancer care, patient outcomes, and system efficiencies. 

## 3. Why Is Timely Access to Molecular Testing Results an Imperative in Canada?

Molecular testing enables accurate identification of a specific tumor profile that indicates that a patient may be a candidate for targeted treatments that are more effective and less toxic. For patients, timely access to molecular testing leads to optimized personalized care that results in improved health outcomes, including clinical, quality of life, and survivorship outcomes [6,7], and by extension lower healthcare costs [8]. For clinicians, biomarker testing helps to predict and monitor response to therapies, increasing clinicians’ ability to prescribe or alter therapy at the right time for a patient to maximize treatment effectiveness, spare unnecessary treatments, and save healthcare resources [9]. Molecular testing enables healthcare providers to avoid providing futile care by negating the use of treatments that are known to be ineffective for a given biomarker profile, thereby reducing risks for toxicity in the absence of benefit as well as unnecessary healthcare costs [10]. Better patient outcomes also alleviate informal caregiver burdens [11], thereby benefiting society as a whole. Biomarker testing also facilitates prognostication, determination of whether a cancer is progressing, and identification of patients with a high risk of recurrence after curative intent therapy [12]. For inheritable cancers, knowing which germline genetic mutations are responsible for the development of a particular cancer may also prompt counselling and/or testing of family members who might benefit from screening or risk-reduction strategies [13]. Finally, large-scale molecular testing allows trends in genomic drivers to be identified, advancing opportunities for cancer research and new drug discovery. In the future, biomarkers will be used to screen for cancers [14]. Overall, timely access to biomarker testing and results improves healthcare outcomes and experiences for patients, their care givers, and their healthcare providers, driving value in the healthcare system as a whole. 

Despite the upfront costs of incorporating molecular testing into care pathways (e.g., costs related to infrastructure, training, human resources), the total cost of using multiple lines of “one size fits all” therapy is actually more costly for the healthcare system. With strategic implementation, over the long-term, advanced molecular testing has the potential to result in overall value for all stakeholders, including system-cost savings [15,16]. While evidence of cost reduction is still being generated, a recent Canadian study investigating the cost-effectiveness of adding circulating tumor DNA (ctDNA or liquid biopsy) to tumor-tissue biopsy testing in clinically selected patients with advanced non-small-cell lung cancer (NSCLC), showed no increase to system costs, while leading to more patients receiving the appropriate targeted treatment [8]. 

Canada is a diverse country with variable access to healthcare for cancer diagnosis and treatment; variability is high within and between provincial jurisdictions [17]. Patients living in rural and remote areas often have restricted access to cancer care compared with those who live in major urban centers. Beyond geography, there are also inequities and disparities in access among patients on the basis of socioeconomic status and race. For example, in Ontario, First Nations people have a higher incidence of many cancers than the general Ontario population, as well as higher mortality from some cancers [18]. A population-based retrospective study in Quebec investigated survival inequities in advanced lung cancer patients by income, education, material deprivation index, immigration, and geographic region, as well as variations in the use and delay of targeted therapy (*EGFR*-TKI treatment). Patients residing in areas with low education, high material deprivation, minimal immigrant density, or in rural areas or urban centers other than Montreal, had shorter median survival times than patients from Montreal [19]. Data from the United Kingdom suggest that as the index of multiple deprivation (a measure of the general poverty of a geographic area) increases, the likelihood of patients receiving any novel anticancer therapy, any targeted therapy, or immunotherapy decreases [20]. Data from the US have shown that, when compared to white patients, African American patients with NSCLC are significantly less likely to receive biomarker testing, and when they do receive testing, are less likely to receive the gold standard next-generation sequencing (NGS) testing [21]. 

## 4. Barriers and Challenges to Biomarker Testing in Canada across Tumor Types

Presentations by patient advocacy groups highlighted the challenges and barriers to biomarker testing in their respective patient populations. The identified challenges situated at the intersection of patients and the healthcare system are summarized by group in Table 1 and are detailed below in a narrative description of common themes and perspectives that emerged from the conference session discourse. 

### 4.1. Lack of Clinician Awareness (Include FPs) of Cancer Biomarkers and Testing Options

Medical knowledge about biomarker testing is highly specialized and rapidly evolving. Before consultation with a medical oncologist, patients typically see a primary care provider and any number of specialists who may have little to no knowledge about cancer biomarkers. In some cases, physicians’ beliefs may be a barrier to biomarking testing. Some oncologists may be focused only on biomarkers directly linked to a specific actionable treatment decision. Less importance may be placed on biomarker testing that is not immediately translatable to a funded treatment option; however, knowledge of other biomarkers can be useful, for instance, in directing patients toward biomarker-driven clinical trials. Clinicians also do not have evidence to take action on results from minimal residual disease (MRD) with ctDNA testing after curative intent therapy for solid tumors. For example, MRD testing is available commercially for breast and colorectal cancer, but it is not being routinely ordered, as the cost for this test is not reimbursed by the Canadian public healthcare system and is currently being investigated through clinical trials to determine if results will guide optimal adjuvant treatment strategies.

### 4.2. Failure by Clinicians to Discuss Biomarkers with Patients

Clinicians may fail to discuss biomarker testing with patients for any number of reasons, including: lack of awareness (as discussed above); a sense that the topic is too complicated to discuss with patients; or a belief that molecular testing findings would not immediately impact the recommended treatment plan. They may also take into account genuine barriers to testing, such as: out-of-pocket costs; lack of private insurance; lack of formal pathways to request testing; and patient preferences to start treatment immediately vs. waiting for biomarker testing results, among others.

### 4.3. Lack of Patient Education and Awareness of Genomic Medicine Options

Patients may trust that their physicians will provide them with all the information they need about their cancers. However, due to the issues outlined above with gaps in physician knowledge and the failure of some physicians to discuss biomarkers with patients, at times, patients are undereducated about their cancer and treatment options [22]. There is a very broad spectrum of understanding and knowledge amongst cancer patients. Patients may not know what a biomarker is, whether their tumor should be or has been tested, or if their biomarker has an actionable treatment. They may not appreciate the differences between inherited germline mutations and somatic mutations that contribute to cancer development. They also may not know if they have the right to ask for testing or if testing is already part of their standard assessments. Further, they may not understand how their biomarkers could impact their treatment options and what expectations they should have.

Consequently, many patients feel that they must seek out information on their own. At a time when they are facing a health crisis, this can translate into significant stress and personal burden that they may wish to avoid. Breast Cancer Canada noted that some patients are turning to online tools developed in the US, only to discuss their findings with their healthcare team and to discover that the options they are interested in are not appropriate for their breast cancer and/or are not available in Canada. This causes further emotional distress.

### 4.4. Lack of Timely Access to Testing and Reporting of Results

Ideally, biomarker testing results should be available at the first meeting with the medical oncologist, but this is often not possible because of delays in availability of results due to long turnaround time. In-house reflex testing (i.e., testing that is ordered automatically by the pathologist) is the gold standard. When samples need to be sent to an outside laboratory, there can be long and variable turnaround times to obtain the results. Oncologists are often the last to be involved in the patient’s diagnostic journey, as patients come to oncology after seeing various other specialists for biopsies or procedures (e.g., colonoscopies). It is critical that these other specialists be aware of downstream impacts of their care, such as collecting quality tissue samples or ensuring that alerts are in place to confirm biomarker testing is ordered to mitigate any delays that may occur when a biomarker test is not ordered until the medical oncologist meets the patient in consultation. This relates back to the issue of healthcare provider awareness described above.

### 4.5. Delays in or No Funded Access to Hereditary Testing

For some hereditary cancers, there is no effective screening tool and no diagnostic test. Identification of those patients at highest risk is imperative in order to offer preventive treatments and ensure appropriate screening protocols are followed. For some cancers in this category, for instance certain ovarian cancers, all patients should have germline testing. In some jurisdictions, there is reflex testing, but in others, patients need to be referred to genetics. Delays in being seen by a genetics team and having germline testing leads to delays in identifying other at-risk members of a family who might benefit from screening or other risk-reduction strategies. Further, targeted therapies are becoming available for certain germline genetic mutations, e.g., *BRCA*. Despite this, there continues to be a lack of national consensus for referrals for genetic counseling in these specific cancer types. 

### 4.6. Intra- and Inter-Provincial Disparities in Access and Funding, and Silos within Institutions and between Provinces

While Canada has a “universal” healthcare system, it is not actually universal in the sense of providing equitable access to care, and is fragmented both within and between provinces. Most provinces do not fund a companion novel diagnostic test at the same time that a new targeted therapy emerges. Within jurisdictions, there are also disparities, often on the basis of geography, where those living in urban centers are more likely to have a biopsy performed in a large academic center with in-house biomarker testing compared with those living in remote rural areas. Further, within the same province, the institutional funding and expertise to perform biomarker testing can vary between sites.

### 4.7. Lack of Funding for Patients to Access Testing

High out-of-pocket costs of biomarker testing preclude access for some potentially eligible patients. The Pancreatic Cancer Action Network (PanCAN) in the US was offering Canadians with pancreatic cancer free access to a biomarker testing program, and in January of 2023 had as many Canadian patients accessing the program as Americans, highlighting the interest and need among Canadians. For tumor types where biomarker testing is not standard of care, patients who do not have private insurance that covers testing costs or who cannot pay out of pocket for the cost of testing, which is often several thousand dollars at minimum, are disadvantaged. For investigational biomarkers and targeted therapies, early phase trials are often very reasonable treatment options for patients with advanced tumors harboring specific molecular aberrations and whose disease has already progressed on standard therapies. Unfortunately, these trials are only options for patients who have the resources to access investigational tumor profiling through institutional translational programs, which are generally located only in a select number of cancer centers with large early phase research programs in major Canadian cities, or even outside of Canada.

### 4.8. Impact of Social Determinants of Health and Stigma

People with lower socioeconomic status are more likely to encounter difficulties in access, self-advocacy, and empowerment. In general, lower rates of health literacy among those with a lower socioeconomic status mean that patients are less likely to be aware of, ask about, and advocate for biomarker testing. Furthermore, lower health literacy is also a significant barrier to clinical trial access, which is another avenue to biomarker testing. The stigma associated with some cancer types, e.g., lung cancer, can lead patients to harbor feelings of self-blame and shame. These feelings make patients less likely to be advocates for the best care for themselves [23].

### 4.9. Lack of Advocacy for Rare Tumors

Patients with rare tumors are among the most disadvantaged, as there may be no support group or organized advocacy voice. In addition, these cancers are less likely to be researched due to challenges conducting large phase III randomized trials. Patients with rare tumors may, therefore, be less likely to have their biomarkers tested due to lack of knowledge or lack of targeted therapies available. The disadvantage of not having an organized advocacy organization is not limited to rare cancers; concrete examples in Canada are endometrial and cervical cancers, two relatively common cancers. While there have been many advances in profiling and treatment selection, to our knowledge, there is no advocacy group to inform patients about the value of biomarker testing. Biomarker-driven therapies have demonstrated overall survival benefit in these gynecologic cancers, yet there are no Canadian patient advocacy groups to champion the advancement of tumor testing and drug access for these patients.

## 5. Molecular Pathology Perspective

Presentations by molecular pathologists highlighted the challenges and barriers to biomarker testing across tumor types. 

### 5.1. Tumor Tissue Testing

The purpose of tumor tissue testing is to evaluate the DNA, RNA, or proteins for mutations that can provide diagnostic, prognostic, or predictive information for disease monitoring and to inform therapy. Which biomarker tests are indicated depends on the cancer type. To test for mutations at the gene level, options include next generation sequencing (NGS), polymerase chain reaction (PCR), or fluorescence in situ hybridization (FISH) testing, while immunohistochemistry (IHC) is used to evaluate biomarkers at the protein level. Tissue samples often pass through multiple laboratories and hands before reaching the molecular laboratory, where there is often much work performed to ensure the sample is evaluable (i.e., that it meets the quality-control thresholds of the relevant assay). 

In an ideal situation, a single test would be able to provide all required biomarker testing, as opposed to having to test for biomarkers sequentially using different techniques, which adds to turnaround times and consumes tissue from what is frequently a limited biopsy sample. This is most likely to be achieved with a broad NGS panel, considered to be the gold standard for biomarker testing for most tumor types. NGS panels that are able to simultaneously test for hundreds of DNA and RNA biomarkers are the basis for programs of comprehensive genomic profiling (CGP). CGP is an approach to assess most, if not all, clinically significant genes, including relevant cancer biomarkers to inform diagnosis, prognosis, and treatment selection, and to monitor therapeutic response.

### 5.2. Circulating Tumor DNA (ctDNA)

Circulating tumor DNA (ctDNA) testing detects genomic information in bodily fluids, primarily cell-free DNA in blood. Also known as liquid biopsy, this technology can be an alternative or complement to traditional tissue biomarker testing. The ability to detect ctDNA has a number of established and emerging utilities, including diagnosing specific mutations that can be treated with targeted therapies. If patients develop progression, especially in an anatomic area that would be difficult or unsafe to biopsy, ctDNA may provide information on the status of the tumor and how the approach to treatment could be best modified. An obvious advantage is requiring only a blood sample, which is easier to obtain than a tissue biopsy and also makes liquid biopsy a potentially more accessible form of biomarker testing for patients in remote/rural locations. It is expected that the technology identifying ctDNA will be used to screen for cancers in the future. It has also been shown to be a powerful prognostic tool for detecting minimal residual disease/early recurrence in cancer patients who have undergone surgery with curative intent [24]. 

### 5.3. Lack of Standardization of Technology, Reporting, Training, and Funding Models

When a biomarker is proposed for funding and for clinical use, it is critical that all laboratories are provided with a standard set of regulations regarding what should be measured (e.g., mutations, insertions and deletions, copy number gains and losses, actionable variants). Currently, these details may be left up to the individual laboratories to determine. An evidence-based guidance document for each tumor type should be available and should be standardized at the provincial level. While some provinces may have frameworks for use within that province, they are not broadly shared or adopted, leading to intra-provincial disparities.

There is significant variation in how molecular diagnostic professionals are trained. Standardized training and practice are needed for reliable testing processes, including data analysis and interpretation. Clinical networks are needed for the purposes of learning and sharing best practices, as interpretation of these tests is not straightforward. In the case of rare mutations, for example, interlaboratory sharing of tissue samples is needed for validation studies. 

There is no standard way that biomarker testing is funded in Canada. Not infrequently, gene-sequencing technologies are readily available, yet there is no pathway for laboratory leaders to purchase these with public funding. Often the funds must come from philanthropic efforts and hospital foundations. While there is funding available to perform certain tests, funding to establish new tests (e.g., *NTRK* rearrangement in various solid tumors) is generally unavailable.

Much of the available technology is concentrated in academic hospitals, yet many patients are treated and reside in community settings. Although some funding goes to academic sites to perform the biomarker testing on outside specimens, there most commonly is no funding for transporting those specimens from a community site to an academic site, or for transmitting that information back from an academic testing site to the community site where the patient and their oncologist are located.

Terminology used to communicate results is another important factor. As noted in the terminology section (Appendix B), there are many terms used to describe biomarker testing. These terms are often used interchangeably, which can lead to confusion and imprecision. This is especially important for pathologists who are receiving biomarker requests and communicating results. It is imperative that biomarker testing and communication be contextualized. For example, the impact of a *BRAF* V600E mutation is quite different in a potentially hereditary cancer syndrome such as Lynch syndrome than in the context of melanoma.

The medical team requesting biomarkers may include the surgeon or the medical oncologist, who must work with the pathologist to clearly communicate what information is needed and when. Requisitions need to provide patient context and details. The rate at which the evidence informing the implications of biomarker findings evolves is rapid and often tumor specific. Thus, biomarker results on pathology reports should include practical interpretation of information to ensure optimal use of that information by the end user responsible for patient care. Centralized documentation in an integrated electronic medical record system is an important enabler of consistent communication and information-sharing among multidisciplinary healthcare teams.

### 5.4. Human Health Resources, Technology Limitations, and Automation

Many laboratories within Canada cannot afford or are not technologically equipped to perform commercially developed companion diagnostic tests approved by regulatory authorities. The handling of tissue and testing is performed by technologists, who are in short supply in many provinces. It is currently difficult to fill positions with appropriately trained personnel, such as technologists, bio-informaticians, molecular pathologists, or medical geneticists, as there is such a strong job market for people with these skills.

One way to address these challenges is with automation, including: machines that extract DNA and RNA from multiple samples at a time; liquid handlers that can perform very rapid automated pipetting needed to create libraries before NGS can be performed; and automated sequencers. In the future, software solutions and artificial intelligence for analyzing and reporting data will play a role.

Recommendations related to data and information infrastructure and health human-resource training requirements are consistent with the necessary conditions that were identified in a recent “A State of Readiness Progress Report” [25].

### 5.5. Reflex Testing

In general, reflex biomarker assessment refers to testing that is triggered in the laboratory by pathologists, without waiting for a request from a clinician. As a more streamlined testing process, there are fewer steps and less cost involved. Turnaround times are improved, meaning it is more likely that complete biomarker information will be available at the time of oncology consultation, facilitating treatment decision making and allowing a patient to be started on treatment in a timely manner. Furthermore, reflex testing standardizes testing for all patients and removes the variability that may occur when different oncologists at the same center do not have the same biomarker-testing ordering practices.

The benefit of the improved turnaround time with reflex testing is not limited to patients with advanced cancer. For instance, a current standard of care for the treatment of early-stage NSCLC is to use neoadjuvant chemotherapy and immunotherapy before surgery; however, this approach is reserved for those patients who have tumors with wild-type *EGFR* and *ALK*. Hence, delayed biomarker results translate into delays in receiving curative intent surgery [26]. Timely reporting can also prevent patients from receiving a more toxic and less effective treatment. For example, for patients with thyroid cancer, enrollment in patient support programs may be the only way to access the best therapy, a multistep process that can be time consuming. These requests are made on case-by-case application process only after biomarker test results are available, and patients who have aggressive disease simply may not have extra time to try to access the best drugs.

### 5.6. Detection of Minimal Residual Disease

Levels of ctDNA will fluctuate depending on response to therapies, and the detection of ctDNA in the absence of clinically detectable tumor burden as a tool to tailor adjuvant therapies and to monitor for disease progression/relapse is under investigation. Large phase III studies, such as the Circulate North America study (open in Canada through the Canadian Clinical Trials Group [CRC10] [27]), are ongoing to determine the role of ctDNA to select the most appropriate adjuvant therapy post-surgery across a variety of tumor types, escalating adjuvant strategies in those who are ctDNA positive. Likewise, these studies are testing the safety of reducing (de-escalating) therapy in those who are found to be at lower risk for future recurrence by virtue of negative MRD testing, ideally defining patient populations that can be spared potentially toxic treatments they do not need. Health economic research is needed to demonstrate that ctDNA testing has the potential to offer value to the system by not wasting resources on treatments with no clinical benefit. 

## 6. Health Technology Assessment

At the policy level in Canada, the current health-technology assessment (HTA) process challenges ease of integration of biomarker testing into the healthcare system. Currently, HTA assessments almost uniformly focus on drug reimbursement, and rarely make recommendations addressing funding for companion diagnostics. HTA funding recommendations are largely made on the basis of phase III clinical trial data along with health-economic analysis of the drug/technology in question. Biomarker-driven care poses a dilemma in the sense that financial decisions will need to be made for increasingly smaller populations and subsets of what may be a common tumor type. Current health technology assessors struggle with that uncertainty, necessitating novel and innovative measures of value. While there has been enormous progress in therapies, there has not been parallel innovation on the health-policy side, and this is impeding access. The inclusion of data collected from a variety of sources beyond clinical trials, including real-world evidence will be important to capture this value through a Canadian lens. Efforts are needed to lobby the decision-making bodies that make up HTA in Canada to adopt a more flexible health-technology approach for rare conditions, to include diagnostic testing, and to enable conditional approvals of medications where there is a defined level of uncertainty (due to the small populations).

## 7. Value-Based Healthcare Transformation

The goal of value-based healthcare (VBHC) transformation is to enable the healthcare system to create more value for patients, while also creating system efficiencies. Indeed, VBHC is seen as a strategy for addressing the challenges facing healthcare, with the underlying idea being that value is created for all stakeholders in the healthcare system when the mutual focus is on improving a person’s health outcomes. Better health equates to reduced consumption of healthcare resources. There is also a component of efficiency, as value is measured in terms of health outcomes achieved relative to healthcare costs expended over the entire trajectory of care, or care-delivery value chain. Hence, the more timely and more effective the care and treatment, the quicker and better the health outcomes achieved, and the lower the need and, therefore, the cost of additional care and/or treatment. Importantly, VBHC defines health outcomes in terms of both clinical and patient-reported outcome measures (PROMs) to reflect a person’s overall health and well-being related to a disease state. The systematic collection of PROMs is required to generate more real-world data and create real-world evidence to inform treatment and public reimbursement decisions, as well as policies directed at improving equitable access to precision medicine in Canada. While measurement and use of PROMSs to inform cancer care are gaining ground in some countries, their adoption is far from systematic in Canada, though some leading patient organizations (e.g., Quebec Cancer Coalition and CCRAN) are forging a path forward in this space. 

In oncology, the patient-care trajectory or delivery value chain can be optimized to improve patient outcomes through integrated care and access to optimal and timely treatment for cancer patients. As described in the sections above, appropriate access to biomarker testing and results offers this opportunity. Comprehensive genomic profiling, in particular, can lead to novel personalized treatment options that have fewer side effects, better health and wellbeing outcomes, and longer survival. Timely access to test results can allow patients to avoid exposure to unnecessary or ineffective treatments that can exacerbate the physical, emotional, and economic burdens of disease. The value of investing in infrastructures that would offer timely and equitable access to biomarker testing and reporting is clear when the VBHC equation is applied. 

## 8. Health System Preparedness for Advanced Biomarker Testing

To better understand Canada’s state of readiness to adopt and implement biomarker testing, a state of readiness report was developed by a group of Canadian researchers [25] to determine the conditions for readiness within three broad categories: (1) infrastructure—the availability of health resources, human resources, and informatics; (2) operations—the ability to implement, coordinate, and evaluate testing at a health system level; and (3) healthcare environment—the healthcare supports such as financing, knowledge translation regarding innovation, education for patients and providers, and regulations. Within these categories, 11 essential conditions were then mapped to Canada’s four largest provinces as well as Nova Scotia, the largest province in Canada’s Atlantic region, to assess their states of readiness for genomic medicine. The following common themes emerged: (1) the need to improve informatic systems and integration of test results into electronic records; (2) the need to be able to identify high-value testing using evaluation processes that adhere to health-technology assessment principles; (3) the need to develop navigational tools to help patients and the general public understand who is eligible for genetic testing and how to access it; and (4) the need for dedicated funding from the public sector (vs. reliance on the private sector) for the development of tests and proficiency testing.

## 9. Call to Action and Recommendations

The following recommendations were generated by the conference experts to address the challenges and barriers to timely biomarker testing access and reporting to ultimately reduce the cancer care inequities in Canada.

(1) Develop and promote targeted oncology education programs, starting with medical school training, to increase physician awareness and understanding of the importance of biomarker testing early in the cancer patient diagnostic journey.
(2) Create biomarker guidance documents to encourage optimal and evidence-based clinical decision-making for every tumor type. From these guidance documents, develop knowledge translation strategies and tools to quickly bring the evidence to the clinical setting.
(3) Provide all cancer patients with a clearly written summary detailing their known biomarker status and what biomarker testing they have received.
(4) Develop patient information and self-advocacy tools for every tumor type (including rare tumors) to guide conversations regarding biomarkers with healthcare providers.
(5) Undertake quality assurance initiatives with the goal of minimizing the number of steps that a sample must go through before results are communicated to patients. Maximize use of automation and AI within pathology laboratories to improve efficiencies in testing.
(6) Implement reflex biomarker testing protocols with the goal of having results available by the time of the first patient oncology visit.
(7) Encourage health technology assessors to consider real-world evidence, based on real-world data and patient-reported outcomes, in their assessments.
(8) Ensure all biomarker testing strategies include measures to mitigate the impact of the social determinants of health, especially in Canada’s most marginalized and remote communities.

## 10. Conclusions

Genomic medicine is a powerful tool to improve diagnosis, prognosis, and quality of life by facilitating the delivery of the right drug at the right dose at the right time for the right patient. More precise and effectual and less toxic treatments are emerging from our understanding of the molecular basis of cancer. Canada must standardize its approach to biomarker testing across the country and prioritize advanced molecular testing to ensure systems are in place to quickly bring innovation and evidence to Canadian cancer patients. Finally, the implementation of biomarker testing must be universal and ensure that no Canadian is left behind due to their place of residence or their socioeconomic status. This biomarker conference brought together patients, patient advocacy organizations, researchers, clinicians, and industry partners to identify the barriers, challenges and opportunities related to equitable access to biomarker testing. These same stakeholders need to continue their individual and joint efforts to develop and implement sustainable solutions.

## Figures and Tables

**Table 1:** Challenges and barriers identified by participating patient organizations.

Tumor Location	Participant Patient Advocacy Organization	Barriers/Challenges/Concerns Identified by Patients and Caregivers
Breast	Breast Cancer Canada	Delays in or no access to hereditary testing Intra- and inter-provincial disparities in access
Colorectal	Colorectal Cancer Resource & Action Network (CCRAN)	Lack of patient education and awareness of genomic medicine options Lack of timely reporting of results Intra- and inter-provincial disparities in access
Gastrointestinal stroma tumor (GIST)	Life Raft Group of Canada	Lack of clinician awareness of genomic medicine options Lack of funding Silos within institutions and between provinces
Lung	Lung Cancer Canada	Lack of patient education and awareness of genomic medicine options Failure by clinicians to discuss with patients Impact of social determinants of health and stigma Intra- and inter-provincial disparities in access
Ovary	Ovarian Cancer Canada	Lack of patient education and awareness of genomic medicine options Failure by clinicians to discuss with patients Intra- and inter-provincial disparities in access
Skin	Save Your Skin Foundation	Lack of timely access to testing Lack of funding for patients to access testing
Pan tumors	Canadian Cancer Survivor Network	Intra- and inter-provincial disparities in access Lack of funding for patients to access testing and for laboratories to provide testing Lack of standardized testing
Pancreas	Craig’s Cause: Pancreatic Cancer Society	Intra- and inter-provincial disparities in access Disparities in funding models Impact of social determinants of health
Stomach	My Gut Feeling	Lack of patient education and awareness Lack of clinician awareness (especially family physicians) Intra- and inter-provincial disparities in access

## Appendix A. Conference Agenda

**Session**	**Speakers**
Day 1 Conference Opening	Dr. Monika Slovinec D’Angelo, chief research officer, CCRAN
Welcome From CCRAN’s President	Ms. Filomena Servidio-Italiano, president and CEO, CCRAN Ms. Emma Langelier, caregiver to metastatic colorectal cancer patient
Why Timely Access to Molecular Testing Results is an Imperative Healthcare Issue in Canada	Dr. Stephanie Snow, medical oncologist, QEII Health Sciences Centre
Patient Access to Precision Medicines: Molecular Testing and Timely Biomarker Reporting (Patient Group Roundtable)	Overview of barriers: Ms. Filomena Servidio-Italiano, president and CEO, CCRAN GIST: Dr. David Josephy, president, GIST Sarcoma Life Raft Group Canada Breast cancer: Ms. Kimberly Carson, CEO, Breast Cancer Canada Lung cancer: Ms. Michele Wright, patient support initiatives coordinator, Lung Cancer Canada Ovarian cancer: Dr. Alicia Tone, scientific advisor and OvCAN project manager, Ovarian Cancer Canada Melanoma: Ms. Kathleen Barnard, President, Save Your Skin Foundation Pan-Canadian tumor perspective: Ms. Lindsay Timm, community engagement manager, Canadian Cancer Survivor Network Pancreatic cancer: Ms. Stefanie Condon-Oldreive, president and CEO, Craig’s Cause Pancreatic Cancer Society Gastric cancers: Ms. Teresa Tiano, chair and co-founder, My Gut Feeling, Stomach Cancer Foundation of Canada Colorectal cancer: Ms. Filomena Servidio-Italiano president and CEO, CCRAN
Podcast with Lung Cancer Caregiver and Advocate Filomena Servidio-Italiano and Dr. Paul Wheatley-Price About Her Uncle’s Inspiring Journey with Stage IV Disease	
Facilitating Access to Precision Therapies for Colorectal Cancer, Lung Cancer, and Thyroid Cancer: Call for Timely Biomarker Reporting	Colorectal cancer perspective: Dr. Sharlene Gill, GI Medical Oncologist, BC Cancer Agency Lung cancer perspective: Dr. Shaqil Kassam, medical oncologist, Southlake Stronach Regional Cancer Centre Thyroid cancer perspective: Dr. Nicole Chau, medical oncologist, BC Cancer Agency Molecular testing perspective: Dr. Brandon Sheffield, pathologist, William Osler Health System
Facilitating Access to Precision Therapies for Breast Cancer, Gastric Cancers, and GIST: Call for Timely Biomarker Reporting	Gastric cancer perspective: Dr. Michael J. Raphael, GI medical oncologist, Odette Cancer Center at Sunnybrook Health Sciences Center Breast cancer perspective: Dr. Christine Brezden-Masley medical oncologist, Mount Sinai Hospital GIST cancer perspective: Dr. Tim Asmis, medical oncologist, The Ottawa Hospital Cancer Centre Molecular testing perspective: Dr. Michael D. Carter, pathologist, Faculty of Medicine, Dalhousie University
Patient Video—Ms. Brenda Clayton, Mother of Young Patient Who Succumbed to Cholangiocarcinoma	
Facilitating Access to Targeted Therapies for Melanoma, Gynecologic Cancers, and Pancreatic Cancer: Call for Timely Biomarker Reporting	Gynecologic cancers perspective: Dr. Neesha Dhani, medical oncologist, Princess Margaret Cancer Centre Melanoma perspective: Dr. Eve St-Hilaire, medical oncologist, Dr-Léon-Richard Oncology Centre, Vitalité Health Network Pancreatic cancer perspective: Dr. Patricia Tang, medical oncologist, Tom Baker Cancer Centre Molecular testing perspective: Dr. Aaron Pollett, anatomic pathologist, Division of Diagnostic Medical Genetics, Mount Sinai Hospital
The Forgotten and Rare Cancers: A Call to Action	Rare cancer perspective: Dr. Michael J. Raphael, GI medical oncologist, Odette Cancer Center, Sunnybrook Health Sciences Center Hepatobiliary cancer perspective: Dr. Ravi Ramjeesingh, medical oncologist and associate professor, Nova Scotia Cancer Centre and Dalhousie University, Chair HPB Oncology Halifax Hematology perspective: Dr. Shannon Jackson hematologist, West Coast Hematology, value-based health care physician lead, Providence Health Care, St. Paul’s Hospital Molecular testing perspective: Dr. Brandon Sheffield, pathologist, William Osler Health System
The Role of Liquid Biopsy Measuring Circulating Tumor DNA (ctDNA) in the Management of Cancer	Dr. Stephanie Snow, medical oncologist, QEII Health Sciences Centre Cancer patient perspective: Mr. Rob L., patient with lived colorectal cancer experience who accessed ctDNA testing Clinical ctDNA expert perspective: Dr. Elena Tsvetkova, medical oncologist, London Health Sciences Centre, Local Principal Investigator, COBRA Study Dr. Vincent Funari, Ph.D., chief scientific officer, Imagia Canexia Industry perspective: Dr. Minetta Liu, MD, chief medical officer, Natera Oncology diagnostics laboratory perspective: Dr. Dennis Dobrovolsky, Ph.D., product manager, Oncology, LifeLabs
Day 1 Closing Remarks	Ms. Filomena Servidio-Italiano, OCT, M.A., president and CEO, CCRAN
Conference Day 2 Opening and Welcome	Dr. Monika Slovinec D’Angelo, chief research officer, CCRAN Ms. Filomena Servidio-Italiano, president and CEO, CCRAN
Health System Preparedness for Advanced Biomarker Testing: Implications for Timely Access to Results	Mr. Fred Horne, health policy advisor, former Alberta health minister Mr. Don Husereau, health economist, School of Medicine, University of Ottawa Dr. Harriet Feilotter, molecular geneticist, Department of Pathology and Molecular Medicine, Queens University Dr. Shantanu Banerji, medical oncologist, CancerCare Manitoba Ms. Suzanne Wood, patient with lived colorectal cancer experience whose care was guided by ctDNA
Optimizing Patient Care Pathways Through Timely Access to Biomarker Test Results	Dr. Monika Slovinec D’Angelo, Ph.D., (moderator) VBHC Expert, chief research officer, CCRAN Ms. Natalie Lipschultz, patient with lived colorectal cancer experience Ms. MJ DeCoteau, founder and executive director, Rethink Breast Cancer Dr. Shannon Jackson, hematologist, West Coast Hematology, Value Based Health Care Physician Lead, Department of Hematology, Providence Health Care, St. Paul’s Hospital Dr. Durhane Wong-Rieger, president and CEO, CORD Dr. Samantha Pollard, senior methodologist, Cancer Control Research, BC Cancer Dr. Ju-Yoon Yoon, molecular pathologist, St. Michael’s Hospital, Unity Health Toronto
Patient Video	
“My Colorectal Cancer Consultant”—A Novel Online Tool Promoting Patient Engagement and Informed Decision Making about Therapeutic Options	Ms. Filomena Servidio-Italiano, president and CEO, CCRAN Dr. Mary De Vera, epidemiologist, assistant professor, University of British Columbia
Clinician Video—Dr. Christopher Lieu, GI Medical Oncologist, University of Colorado Hospital	
The Utility and Value of Comprehensive Genomic Profiling (CGP) in Cancer Care	Cancer patient perspective: Mr. Allen Chankowsky, rare-cancer patient with lived experience U.S. CGP research and program perspective: Dr. Razelle Kurzrock, professor of medicine, associate director of clinical research; Linda T. and John A. Mellowes, chair of precision oncology, MCW Cancer Center and Center for Genomic Sciences and Precision Medicine; founding director, Michels Rare Cancers Research Laboratories, Froedtert and MCW Canadian CGP research and program perspective: Dr. Howard Lim, medical oncologist, B.C. Cancer Agency Value-based healthcare perspective: Ms. Eva Villalba, MBA VBHC Expert, executive director, Quebec Cancer Coalition
What Will it Take to Adopt Comprehensive Genomic Profiling (CGP) as a Standard of Cancer Care in Canada?	Mr. Don Husereau, health economist, School of Medicine, University of Ottawa Patient perspective: Dr. Marko Yurkovich, primary care physician and stage IV colorectal cancer 4 patient Clinical practice perspective on CGP in cancer care: Dr. Cathy Eng, medical oncologist and professor, Vanderbilt-Ingram Cancer Center, Nashville, TN, U.S. CGP access perspective (Canada): Dr. Faisal M. Khan PhD, D(ABHI), director, Molecular Diagnostics, OncoHelix NGS technology perspective: Dr. Jeffrey M. Smith, NGS precision medicine director, Clinical Next-Generation Sequencing and Oncology Division, Thermo Fisher Scientific CGP access perspective (U.S.): Dr. John L. Fox, MD MHA senior medical director for the Americas, Market Access, Illumina
Closing Remarks	Ms. Filomena Servidio-Italiano, OCT, M.A. president and CEO, CCRAN
**Time** **Session Title**	**Speakers**
Conference Day 2 Opening and Welcome	Dr. Monika Slovinec D’Angelo, chief research officer, CCRAN Ms. Filomena Servidio-Italiano, president and CEO, CCRAN
Health System Preparedness for Advanced Biomarker Testing: Implications for Timely Access to Results	Mr. Fred Horne, health policy advisor, former Alberta health minister Mr. Don Husereau, health economist, School of Medicine, University of Ottawa Dr. Harriet Feilotter, molecular geneticist, Department of Pathology and Molecular Medicine, Queens University Dr. Shantanu Banerji, medical oncologist, CancerCare Manitoba Ms. Suzanne Wood, patient with lived colorectal cancer experience whose care was guided by ctDNA
Optimizing Patient Care Pathways Through Timely Access to Biomarker Test Results	Dr. Monika Slovinec D’Angelo, Ph.D., (Moderator) VBHC Expert, chief research officer, CCRAN Ms. Natalie Lipschultz, patient with lived colorectal cancer experience Ms. MJ DeCoteau, founder and executive director, Rethink Breast Cancer Dr. Shannon Jackson, hematologist, West Coast Hematology, Value Based Health Care Physician Lead, Department of Hematology, Providence Health Care, St. Paul’s Hospital Dr. Durhane Wong-Rieger, president and CEO, CORD Dr. Samantha Pollard, senior methodologist, Cancer Control Research, BC Cancer Dr. Ju-Yoon Yoon, molecular pathologist, St. Michael’s Hospital, Unity Health Toronto
Patient Video	
“My Colorectal Cancer Consultant”—A Novel Online Tool Promoting Patient Engagement and Informed Decision Making about Therapeutic Options	Ms. Filomena Servidio-Italiano, president and CEO, CCRAN Dr. Mary De Vera, epidemiologist, assistant professor, University of British Columbia
Clinician Video—Dr. Christopher Lieu, GI Medical Oncologist, University of Colorado Hospital	
The Utility and Value of Comprehensive Genomic Profiling (CGP) in Cancer Care	Cancer patient perspective: Mr. Allen Chankowsky, rare-cancer patient with lived experience U.S. CGP research and program perspective: Dr. Razelle Kurzrock, professor of medicine, associate director of clinical research; Linda T. and John A. Mellowes, chair of precision oncology, MCW Cancer Center and Center for Genomic Sciences and Precision Medicine; founding director, Michels Rare Cancers Research Laboratories, Froedtert and MCW Canadian CGP research and program perspective: Dr. Howard Lim, medical oncologist, B.C. Cancer Agency Value-based healthcare perspective: Ms. Eva Villalba, MBA, VBHC expert, executive director, Quebec Cancer Coalition
What Will it Take to Adopt Comprehensive Genomic Profiling (CGP) as a Standard of Cancer Care in Canada?	Mr. Don Husereau, health economist, School of Medicine, University of Ottawa Patient perspective: Dr. Marko Yurkovich, primary care physician and stage IV colorectal cancer 4 patient Clinical practice perspective on CGP in cancer care: Dr. Cathy Eng, medical oncologist and professor, Vanderbilt-Ingram Cancer Center, Nashville, TN, U.S. CGP access perspective (Canada): Dr. Faisal M. Khan PhD, D(ABHI), director, Molecular Diagnostics, OncoHelix NGS technology perspective: Dr. Jeffrey M. Smith, NGS precision medicine director, Clinical Next-Generation Sequencing and Oncology, Division, Thermo Fisher Scientific CGP access perspective (U.S.): Dr. John L. Fox, MD MHA senior medical director for the Americas, Market Access, Illumina
Closing Remarks	Ms. Filomena Servidio-Italiano, OCT, M.A. president and CEO, CCRAN

## Appendix B. Conference Terminology

For the purposes of conference, the following broadly defined terms were used:

Biomarkers (biological markers)—Genes, proteins and other biological molecules that can be measured objectively. Biomarkers can represent a cancer’s unique “fingerprint” and provide diagnostic, prognostic, or predictive information related to treatment response. Also referred to as: tumor testing, tumor genetic testing, genomic testing, genomic profiling, molecular testing, molecular profiling, somatic testing, or tumor subtyping.

Genomic medicine—The use of laboratory-based biomarkers that measure the expression, function, and regulation of genes and gene products to aid healthcare decision making and scientific discovery.

Genomic profiling—A laboratory method that uses a sample of tissue, blood, or other body fluid to learn about all the genes in a person or in a specific cell type, and the way those genes interact with each other and with the environment. Genomic profiling may be conducted to find out why some people get certain diseases while others do not. Genomic profiling may also be conducted on tumor tissue to look for mutations or other genetic changes in a tumor’s DNA. Also referred to as: advanced testing, genetic testing, and genome-based testing.

Next-generation sequencing (NGS)—An advanced technology that permits the sequencing of up to millions of fragments of DNA or RNA in parallel to determine genetic variations associated with cancer. More appropriately called massively parallel sequencing.

Omics-based sequencing technologies—Precision-medicine technologies based on whole-genome (the entire DNA code of an individual), exome (the DNA code of all protein-coding regions), transcriptome (global gene expression as measured by RNA), and proteome (the protein expressed by a cell) sequencing. They characterize the complete or comprehensive set of molecular information of individuals’ cells and tissues. This approach is in contrast to targeted approaches that examine a limited number of genes, genomic areas, or proteins.

Precision medicine/precision oncology—An approach to tailoring disease prevention and treatment that takes into account differences in people’s genes, environments, and lifestyles.

Whole genome sequencing—Considers all of the 20,000 genes in the body (human genome), compared to targeted gene sequencing, which examines a subset of 100 to 500 genes that are most likely to have been mutated.
